# Virulence determinants of MRSA causing skin and soft tissue infections among HIV patients from Chennai

**DOI:** 10.1186/1471-2334-14-S3-E44

**Published:** 2014-05-27

**Authors:** Padma Krishnan, A Nagarajan, J Suriakumar

**Affiliations:** 1 Department of Microbiology, Dr. ALM PGIBMS, University of Madras, Chennai, India; 2 Food Analysis Laboratory, TNFSDA, Madurai, India; 3 Govt. Hospital of Thoracic Medicine, Tambaram, Chennai, India; 4 Institute of Microbiology, Madurai Medical College, Madurai, India

## Background

MRSA is a well armed pathogen with an array of virulence factors, which causes skin and soft tissue infections (SSTIs) among healthy individuals in community. Although there are few reports on MRSA causing SSTIs among HIV patients, there are no studies on their virulence determinants. Hence, the current study was done to detect the virulence determinants of MRSA causing SSTIs among HIV patients.

## Methods

A total of 70 clinical isolates of *Staphylococcus aureus* collected from HIV patients with SSTIs were screened for MRSA using cefoxitin disc (30µg). Detection of *pvl*, *femA*, *mecA* of the isolates was done by triplex PCR. The enterotoxins A–E, J, K, P and egc cluster, exfoliatins A,B and D, hemolysins α, β, γ, δ & γv, leucocidins lukDE & lukM and innate immune evasions were detected by using multiplex PCRs using suitable *S. aureus* controls.

## Results

Of the 70, 16 (22.85%) of the *S. aureus* isolates causing SSTIs among HIV patients were found to be MRSA. *pvl* was detected in 15/16(93.75%) of MRSA isolates. 14/22(58.33%) virulence determinants tested were detected among the MRSA isolates. Among enterotoxins, *sea* and *egc* gene cluster were found in all MRSA isolates tested. 11/16(68.75%) and 10/16(62.50%) MRSA isolates were found to be positive for *chp* and *lukD-E*, respectively.

## Conclusion

Although the MRSA percentage among HIV patients was low, prevalence of *pvl* and other virulence determinants was found to be high among MRSA isolates causing SSTIs in HIV patients.

